# Silent Scars in the Water–Energy–Food Nexus: How Resource Insecurity Shapes Women’s Mental and Reproductive Health in South Africa

**DOI:** 10.3390/ijerph23020187

**Published:** 2026-01-31

**Authors:** Lucy Khofi, Blessings Nyasilia Kaunda-Khangamwa, Andisiwe Maxela, Emily Ragus, Sylvester Mpandeli

**Affiliations:** 1 School of Public Health, University of the Witwatersrand, Johannesburg 2193, South Africa; 2 Department of Anthropology, University of Amsterdam, 1001 NA Amsterdam, The Netherlands; 3 School of Global and Public Health, Kamuzu University of Health Sciences, Blantyre P/Bag 360, Malawi; 4 Department of Sociology, Anthropology, and Development, University of Fort Hare, Alice 5700, South Africa; 5 Water Research Commission of South Africa (WRC), Lynnwood Manor, Pretoria 0001, South Africa

**Keywords:** water supply, energy poverty, food insecurity, gender based violence, mental health, reproductive health, qualitative research, South Africa

## Abstract

**Highlights:**

**Public health relevance—How does this work relate to a public health issue?**

- This study shows how water, energy, and food scarcity directly shape women’s mental and reproductive health in South Africa.
- It highlights the links between resource insecurity, intimate partner violence, unsafe abortion, menstrual health challenges, and psychological distress.

**Public health significance—Why is this work of significance to public health?**

- The findings demonstrate that the Water–Energy–Food nexus is a determinant of health, not only an environmental or technical concern.
- The study provides rare qualitative evidence on embodied and emotional impacts of scarcity, addressing a critical gap in WEF, gender, and health research.

**Public health implications—What are the key implications or messages for practitioners, policy makers and/or researchers in public health?**

- Integrated policies must link water, energy, and food provision with reproductive health, mental health, gender-based violence protection, and social support.
- Addressing scarcity requires gender-responsive, rights-based approaches that protect reproductive autonomy, reduce survival sex, and build structural care systems.

**Abstract:**

Women in resource-scarce communities navigate daily scarcity, structural neglect, and gendered violence, leaving profound but often invisible impacts on mental and reproductive health. Women play an active role in the Water–Energy–Food (WEF) space; they provide water, food, and household security daily. This study investigates how chronic deprivation across the WEF nexus shapes experiences of psychological distress, reproductive vulnerability, and social marginalization in South African settings: Lorentzville, a migrant urban informal settlement, and Mqanduli, a peri-urban Eastern Cape community. Using ethnographic methods, including in-depth interviews, focus group discussions, and participatory observation, and an analytical framework combining structural violence and feminist political ecology, we show that insecurity over water, energy, and food constrains reproductive autonomy, amplifies self-reported symptoms of anxiety and depression, and drives coping and adaptation strategies such as informal work, transactional sex, and fragile social support networks. These strategies, while mitigating immediate risks, cannot fully offset systemic harms. By foregrounding women’s lived experiences, this study extends the WEF nexus framework to include embodied, emotional, and reproductive dimensions, linking historical legacies of colonial and apartheid neglect to contemporary inequities. The findings offer critical insights for integrated health, social, and resource policy interventions that center on gender, care, and justice within environmental, wellbeing, and livelihood.

## 1. Introduction

Access to clean water, reliable energy, and sufficient food is foundational for human health, yet millions of women in low- and middle-income countries experience chronic deprivation that profoundly affects their physical, mental, and reproductive wellbeing [1]. The Water–Energy–Food (WEF) nexus is a conceptual framework that highlights the interconnections and interdependencies among these critical resources, emphasizing that actions in one domain (e.g., water management) can have direct and indirect consequences on energy availability and food security, and vice versa [2,3]. The WEF nexus has been conceptualized in such a way that it should promote interlinkages, create trade-offs and assist sectors and communities to sustain our natural resources. While the WEF nexus has been widely adopted in policy and sustainability discourse, much of the literature remains technocratic, focusing on efficiency, resource management, and sustainability goals while neglecting the lived realities, gendered experiences of scarcity [1]. However, it is important to point out that one of the WEF nexus strengths is to break the “silo “mentality or approach and promote nexus thinking [1,2,3,4,5].

In practice, women disproportionately bear the burden of securing water, water fetching, cooking fuel, and food, often under conditions of poverty, social marginalization, and gendered violence, rendering them particularly vulnerable to cumulative forms of harm [1,5]. Chronic deprivation across the WEF nexus is not merely an environmental, physical, or logistical problem but a deeply embodied form of structural violence, leaving silent scars on women’s mental and reproductive health. Scarcity is experienced through everyday practices: fetching unsafe water, cooking over firewood, pellets, charcoal, or paraffin/kerosene in poorly ventilated spaces, or rationing limited food supplies [1,5,6]. It is unfortunate that women are expected to perform above roles while men are making decisions on behalf of these women. These experiences are intertwined with broader social inequalities, including urban, peri-urban, and informal settlement conditions, rural infrastructural neglect, and systemic discrimination in healthcare and social services [7,8,9]. By placing women’s lived experiences at the center, this study expands the WEF nexus framework, positioning mental and reproductive health as integral indicators of resource justice.

Linking the WEF nexus to reproductive justice, Ross & Solinger [10] highlight that women’s ability to control their reproductive lives, deciding if, when, and how to have children, is inseparable from access to resources and social protections [10]. Water, energy, and food insecurity directly constrain reproductive autonomy, from limiting menstrual hygiene and contraception use to heightening risks of unsafe abortion and coerced sexual labor to find food or resources for their livelihood [1,11]. By connecting resource access with reproductive choice, this study demonstrates that sustainable development and gender equality cannot be achieved without addressing embodied inequities within the WEF nexus.

Globally, evidence indicates that women’s reproductive and mental health are closely linked to resource insecurity [12,13]. In Kenya, Malawi, and Tanzania, water scarcity, water fetching not only increases stress, but compromises menstrual hygiene, sanitation and heightens vulnerability to sexual coercion [5,14,15]. In urban India, energy poverty correlates with domestic violence and psychological distress [16]. Yet, most WEF studies in sub-Saharan Africa and beyond remain empirically thin, lacking rich qualitative evidence on how scarcity interacts with social and gendered inequities [1,5,12,13,16,17], a gap this study addresses.

Guided by structural violence theory, Farmer [18] and feminist political ecology by Elmhirst [19,20], this research examines how systemic deprivation produces cumulative physical, emotional, and reproductive harms. Structural violence conceptualizes harm as embedded in social, political, and economic structures, where poverty, inequality, and state neglect translate into chronic suffering. Feminist political ecology highlights the gendered dimensions of environmental access and control, emphasizing how women’s labor and bodily experiences are inseparable from ecological and resource dynamics [19,20]. By integrating these frameworks, the study captures how scarcity is socially produced, experienced corporeally, and resisted through everyday strategies.

Specifically, this study addresses three interrelated objectives. First, it explores how water, energy, and food insecurity shape women’s reproductive and mental health. Second, it examines how women navigate survival strategies, including informal work, transactional sex, and fragile social networks, under chronic scarcity. Third, it analyses how structural, institutional, and historical inequities, stemming from apartheid, colonial legacies, and contemporary governance failures, exacerbate vulnerability and constrain women’s reproductive autonomy. The study draws on ethnographic methods, including in-depth interviews, focus groups, and participatory observation, conducted in two contrasting South African sites: Lorentzville, an urban migrant settlement in Johannesburg, and Mqanduli, a peri-urban community in the Eastern Cape.

By foregrounding women’s narratives, this study extends the Water, Energy, Food nexus beyond its conventional technocratic framing and positions it within the social, political, and emotional realities of everyday life. Rather than viewing scarcity as a technical failure, the framework conceptualizes it as a gendered condition shaped by structural inequality, historical marginalization, and overlapping forms of exclusion. This orientation aligns with feminist political ecology, which argues that resource systems must be understood through the lived experiences of those who depend on them [19,20].

**Figure 1:**
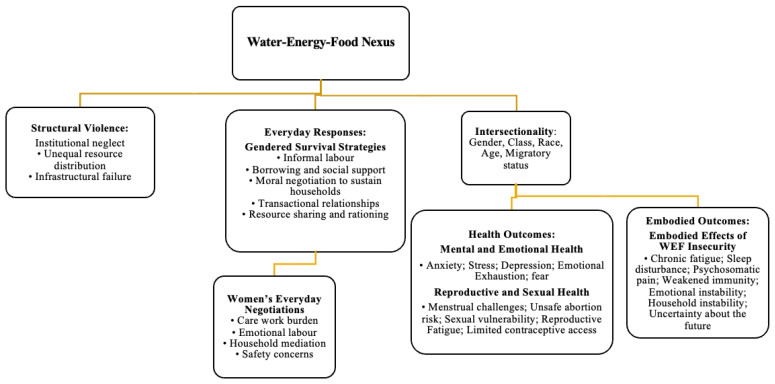
Expanded WEF Nexus framework.

Figure 1 presents the expanded WEF Nexus framework that guides the analysis. The model illustrates how water, energy, and food insecurities interact to produce conditions of heightened vulnerability for women. These insecurities are mediated by structural violence, which operates through absent or failing institutions, unequal resource distribution, and the cumulative effects of poverty and social neglect [18]. Intersectionality further shapes women’s experiences by revealing how class, race, age, migratory status, and household position intensify constraints on autonomy and access [21,22,23,24].

Within this context, women engage in a range of everyday practices that function as survival strategies. These include informal labor, borrowing, negotiating household roles, and, at times, entering transactional relationships to secure basic needs. These strategies reflect agency and resilience, yet they also expose women to emotional strain, reproductive risk, and mental distress. The framework conceptualizes reproductive health and mental health as interconnected outcomes of resource scarcity rather than separating them into isolated categories. Each represents a dimension of embodied experience through which deprivation becomes visible, from fatigue and stress to unsafe abortion, menstrual challenges, fear, and anxiety.

Importantly, the model recognizes that scarcity is not only experienced as harm, but also as a site of negotiation where women continuously exert effort to sustain their households. This approach shifts the focus from victimhood to the creative and constrained forms of agency that women mobilize daily, even within oppressive structural conditions. In doing so, it highlights the moral negotiations women undertake to maintain dignity, nurture families, and preserve social relationships despite systemic failure.

The framework, therefore, positions WEF insecurity as a relational and political condition that shapes women’s bodies and emotions and influences their reproductive and mental well-being. It provides the analytical basis for interpreting the empirical results presented later in the manuscript.

## 2. Materials and Methods

This article draws from a broader ethnographic study conducted between 2022 and 2024 in two South African communities, Lorentzville in Johannesburg and Mqanduli in the Eastern Cape. The larger project explored how water, energy, and food insecurities shape everyday life, gender relations, and bodily wellbeing. For this manuscript, the analysis focuses specifically on the thirty-six women whose narratives offer detailed insights into the intersections of material deprivation, reproductive health, and mental well-being. The study adopted a participatory ethnographic design that recognizes lived experience as a form of situated knowledge and political insight [24,25,26]. This approach positions participants as co-constructors of meaning and illuminates the constrained choices and actions that reflect how structural inequality becomes embodied [27,28,29,30,31].

Fieldwork was carried out over an 18-month period. Both Lorentzville and Mqanduli were purposively selected to reflect contrasting socioecological conditions. Lorentzville, an inner-city Johannesburg settlement, is characterized by informal housing, migrant diversity, and precarious access to municipal services, whereas Mqanduli represents a peri-urban context where women rely on communal gardens, rain-fed agriculture, and local governance structures to sustain households [32]. The comparative design allowed for the tracing of how structural neglect manifests differently across peri-urban and urban spaces, while centering women’s experiences as analytical entry points into broader systems of inequality.

Data collection integrated participant observation, in-depth interviews (IDIs), focus group discussions (FGDs), informal dialogs, and document review to enable methodological triangulation [33]. The first author conducted sustained participant observation in both sites, attending council meetings, community garden sessions, and household food distribution activities in Mqanduli and engaging in collaborative relief and recycling initiatives in Lorentzville. These activities fostered mutual trust and offered embodied insight into the rhythms of women’s labor, care practices, and survival strategies. Participant observation was essential for understanding how people live with and through scarcity, rather than only what they say about it, a hallmark of lived experience methodologies [34,35].

A total of 36 in-depth interviews with women were included in this analysis, drawn from the broader dataset collected across Lorentzville and Mqanduli. These interviews ranged between 60 and 90 min and focused on women’s experiences of water, energy, and food access, caregiving responsibilities, reproductive health challenges, and emotional distress. While the wider project also generated additional interviews and focus group discussions, this paper intentionally centers the narratives of these 36 women because they provide the richest and most detailed accounts of how resource insecurity intersects with gendered labor, survival strategies, and health. This approach allows for a focused, in-depth examination of the embodied and emotional dimensions of WEF scarcity within the two study sites.

All audio recordings were transcribed verbatim, translated where necessary, and anonymized. Fieldnotes were typed and integrated with transcripts for analysis. Data analysis followed Braun and Clarke’s [36] six-phase approach to thematic analysis, combining deductive themes informed by theoretical frameworks (e.g., gendered resource labor, structural neglect, embodied suffering) with inductive codes emerging from women’s narratives (e.g., fear, shame, resilience, solidarity). Coding was iterative and reflexive, with the researcher moving back and forth between data and theory to ensure analytic depth [37,38]. To enhance rigor, emergent themes were reviewed with supervisors and cross-checked against fieldnotes. Triangulation across methods and sites strengthened credibility, while thick description and reflexive engagement enhanced transferability and confirmability [39,40].

Triangulation was achieved by comparing insights from interviews, focus groups, participant observation, and fieldnotes. These data sources informed one another and strengthened theme development by allowing cross-checking of emerging patterns across methods and sites.

Reflexivity was central to ensuring methodological integrity throughout the generation and analysis of the 36 interviews included in this paper. The first author, a medical anthropologist with a longstanding engagement in gender and climate justice work, occupied an insider-outsider positionality that shaped data collection and interpretation. In Mqanduli, linguistic and cultural familiarity facilitated rapport and supported deeper exploration of sensitive topics. In Lorentzville, her position as a relative outsider required a slower process of trust building, including accompaniment by community volunteers and sustained participation in communal activities. Reflexive journaling, analytic memos, and regular supervisory discussions helped examine how positionality, emotion, and power relations influenced coding decisions and theme development [41,42].

Trustworthiness was ensured through prolonged engagement in both sites, reflexive journaling, detailed fieldnotes, and supervisory debriefings. Credibility was supported through triangulation and the use of thick, contextually grounded descriptions. Transferability was enhanced through detailed accounts of the study settings and participant characteristics. Dependability was supported by maintaining careful documentation of analytic processes and decision-making. Confirmability was strengthened through reflexive practice and the creation of an audit trail that recorded analytic reflections and methodological choices.

Ethical principles of autonomy, confidentiality, and beneficence guided the study. Ethical clearance was obtained from the University of the Witwatersrand Human Research Ethics Committee (Medical) (Ref: M220760). Participants provided written informed consent prior to participation and were assured of the voluntary nature of their involvement and the right to withdraw at any stage. Given the sensitivity of topics such as intimate partner violence and reproductive health, participants were provided with information on psychosocial and health referral services. All identifying information was removed during transcription to protect anonymity.

Ultimately, this methodological approach foregrounded lived experience as epistemology, that is, as a way of knowing grounded in daily struggle, care, and resilience [43]. By engaging deeply and relationally with participants, the research aimed to reveal the entanglement of structural and emotional worlds, showing how women’s narratives of water fetching, cooking with firewood, pellets, charcoal, or paraffin, food provision, and reproductive care illuminate the broader political ecology of inequality in South Africa.

## 3. Results

Across Lorentzville and Mqanduli, women’s lived experiences of water, energy, and food scarcity were deeply intertwined with mental and reproductive health vulnerabilities. Scarcity shaped emotional, physical, and social dimensions of daily life, producing stress, anxiety, and somatic symptoms, while constraining reproductive autonomy and forcing survival strategies that often included transactional or coerced sexual activities. Women’s coping strategies, ranging from informal work to collective care networks, provided limited relief but could not offset systemic neglect. These findings illustrate the compounding effects of structural deprivation, gendered labor, and social marginalization on women’s wellbeing, highlighting the silent scars embedded in WEF insecurity.

Table 1 and Table 2 below present the sociodemographic characteristics of the women interviewed in Lorentzville and Mqanduli. These tables highlight age, marital status, migration and citizenship status, education level, and primary survival strategies, providing essential context for understanding the vulnerabilities described in the thematic analysis.

The sociodemographic characteristics outlined in Table 1 and Table 2 provide the foundation for interpreting the findings that follow. They show how age, marital status, migration history, education level, and income shaped women’s exposure to scarcity, reproductive vulnerability, and mental distress in each setting. With this context established, the thematic analysis below examines how women described living within scarcity, negotiating fragile solidarities, carrying gendered labor burdens, navigating reproductive and sexual vulnerability, and experiencing the slow violence of structural neglect (see Table 3, below).

### 3.1. Living Within Scarcity: The Emotional Texture of Everyday Survival

Based on the analysis from both Lorentzville and Mqanduli, scarcity shaped daily life in profoundly emotional and bodily ways (see Table 3). Participants described a relentless, grinding pressure that dictated not only what they ate, but how they felt, moved, and made decisions. All mental health references in this section reflect self-reported emotional and physical distress described by participants rather than clinically assessed diagnoses. In Lorentzville, women often began their days before sunrise, queuing for water at communal taps that sometimes ran dry by midmorning. Nelly, 55, described her morning routine:

“I wake before the sun rises, carrying empty containers to the tap. Sometimes I queue for an hour, sometimes two. By the time I get home, my legs are shaking, my back aches, and I have to decide whether to cook with what little I have or wait for the kids to finish school and hope they bring something from the neighbor. Every day feels like a battle I cannot pause, and when I lie down at night, I replay every choice, every penny I couldn’t stretch, every meal I had to skip.”

Load-shedding and illegal electricity cuts compounded these challenges. By afternoon, cooking, refrigeration, and household safety were frequently disrupted. Maria, 43, articulated the cognitive and emotional toll:

“When I lie down, my mind does not stop. I count what is left of the maize meal, the candles, and I count the debts. My heart jumps like a drum. I cannot sleep because tomorrow, I must start again.”

Women described “thinking too much” (ukucabanga kakhulu) or feeling that “my mind is burning”, expressions that captured anxiety, overthinking, and physical exhaustion. Headaches, insomnia, and palpitations were commonly reported, illustrating how scarcity becomes an emotional and somatic burden. Most participants across both sites described this pervasive cognitive fatigue, indicating that living within scarcity is not only a practical struggle but a sustained emotional labor.

In Mqanduli, the landscape of scarcity was peri-urban and infrastructural. Distances between boreholes, irregular rainfall, without access to electricity and transport costs created daily cycles of exhaustion. Asanda, a mother of three, reflected:

“Sometimes I just sit outside and look at the hills. I feel empty. I don’t cry anymore. Even my tears are tired. I try to sleep, but I wake, thinking about what I cannot do for my children.”

Participants normalized these experiences as “part of being a woman,” revealing gendered expectations of endurance. Over time, this normalization blurred the boundaries between hardship and pathology, creating the slow violence of despair. Women in both settings emphasized that scarcity is not merely material; it is deeply relational and embodied, shaping social roles, family responsibilities, and self-perception. This leaves emotional scars on the majority of women in different communities; it is a societal problem.

### 3.2. Fragile Solidarities and Collective Care

Despite pervasive hardship, women forged networks of solidarity and mutual support. In Lorentzville, informal savings clubs (stokvels), shared cooking, gardening, and queuing together for water created small pockets of relief. Josephine described:

“We laugh in line, so we don’t cry. Sometimes joking is the only way to stay alive. When someone has nothing, we all give what we can: a little maize, a candle, a potato. In those moments, we forget hunger for a while.”

In Mqanduli, women gathered for collective gardening, evening prayers, or food-sharing. Zanele, 36, said:

“We share what little we have. When someone loses hope, we visit her house and cook together. I remember one night, a neighbor had nothing for her children. We all brought what we could: maize, tomatoes, and a little oil. We sat together on the floor, and for a while, it felt like there was no hunger. We laughed, we cried. In those moments, I forget the government, I forget the pain outside. This is how we keep going.”

While these solidarities provided critical emotional and material support, participants emphasized their fragility. Competition for scarce resources, exhaustion, and conflict occasionally eroded social ties. Yet, most women agreed that these networks were essential for survival, demonstrating how relationality and care persist even under structural neglect.

### 3.3. Gendered Labor and Emotional Exhaustion

Scarcity emerged as a distinctly gendered labor, a daily choreography of care, planning, and negotiation. Women carried the moral responsibility for feeding, washing, and keeping the household functioning, often invisibly and without support. Josephine, 41, from Lorentzville, captured the emotional weight:

“People say we are strong, but inside, I am always scared. If I stop planning, the children will go hungry. I make lists in my head, debts, shopping, who owes me what, and how much I can borrow. I smile in front of everyone, but at night I cry. Sometimes I whisper to myself, ‘Why me? Why always me?’ Even when I laugh with the neighbors, it feels like my heart is holding a storm.”

In Mqanduli, patriarchal norms intensified emotional labor. Women were excluded from community decisions, land allocation, and water management, yet remained responsible for daily sustenance. Nozipho, 29, explained:

“Men decide, women fix. We speak softly because if you question, they say you are disrespectful. I carry the buckets, fetch the food, and pray that the children are fed. The work is endless, but we cannot stop.”

Many women reported periods of social isolation, loss of appetite, and feelings of mental collapse, a state described as “losing the mind.” Across participants, these experiences indicated that emotional labor under scarcity is both invisible and cumulative, masking the state’s absence in care provision while sustaining household survival. The feminization of scarcity emerges not just as a material condition but as an affective regime that structures everyday life.

### 3.4. Reproductive Pain, Sexual Violence, and Constrained Autonomy

Scarcity extended into the reproductive and sexual sphere, directly impacting women’s health and autonomy. Women reported menstrual infections, miscarriages, and reproductive exhaustion linked to inadequate water, sanitation, and nutrition. Nokuthula, 37, described the challenge of maintaining hygiene in Lorentzville:

“I bleed, and it burns. The clinic says keep clean, but how? The toilets are locked, and the water is for drinking. I wash in buckets, but it is never enough. I feel my body failing me.”

In Mqanduli, reproductive strain was tied to overwork and poor nutrition. Thandi, 25, recounted a miscarriage:

“I had been fetching water for the whole morning, carrying buckets on my head. My body ached, and I thought maybe the baby would be okay. But at night, I felt no movement, nothing. At the clinic, they said I overworked. But if I rest, who fetches water? Who cooks? Who feeds the children? That night, I felt the baby stop moving. I cried alone, because if I cried in front of anyone, they would tell me to be strong, to endure. But inside, I was broken.”

Access to contraception and abortion reflected stark inequities. Undocumented women in Lorentzville often faced outright denial of services. Grace, a Malawian woman, shared:

“I went to the clinic, but they said I need papers. I tried to buy the pills from someone on the street. I took them, and I bled heavily. I fainted once, woke up scared to go back. Every time I thought of going back, I remembered how they judged me the first time. It felt like my body was not mine anymore.”

In Mqanduli, young women faced moral policing by nurses, restricting access to contraception and safe abortion. Nontombi, 22, reflected:

“They say we are too young to use pills, but when we get pregnant, they call us irresponsible. I feel trapped. The clinic is supposed to help, but it only judges me.”

Most participants echoed these experiences, linking reproductive vulnerability to structural neglect, scarce resources, and gendered power relations. Unsafe abortions, contraceptive barriers, and miscarriage trauma were further compounded by anxiety, depression, and fear of intimate partner violence (IPV), showing the inseparability of reproductive and mental health under scarcity.

### 3.5. Intersections of Scarcity, Violence, and Despair

Scarcity and gender-based violence were tightly intertwined. In Lorentzville, economic dependence on male partners made it nearly impossible to leave abusive relationships. Phindile, 32, explained:

“He beats me when he drinks, but I stay. If I go, who will feed the children? I can handle the pain, but not hunger. Every day, I pretend I am fine, but at night, I feel my body breaking.”

In Mqanduli, male frustration over unemployment and scarcity often escalated into violence. Luleka, 40, said:

“When there is no food, he blames me. He says I make him feel useless. Then he hits me. I hide until he cools down. Sometimes I cannot leave the house for days.”

Some women engaged in transactional sex as survival strategies to access food, rent, or electricity. Tebogo, 26, shared:

“If I say no, he cuts the power. If I say yes, I hate myself. I try to smile in front of the children, but at night I cannot sleep. Sometimes I hit my pillow and scream silently, because nobody should see the shame. I feel trapped. My body pays the price for hunger and fear.”

These narratives illustrate how scarcity, IPV, and survival sex intersect into a continuum of structural and interpersonal violence. Emotional exhaustion, panic attacks, and numbing were widely reported. Most participants described these experiences as a ‘sickness of the soul,’ reflecting the psychosocial toll of enduring persistent scarcity and risk.

### 3.6. The Slow Violence of Neglect

Participants framed their suffering as a consequence of political abandonment rather than misfortune. Nelly, 55, in Lorentzville, reflected:

“This government kills us slowly. Not with bullets, but with waiting. Waiting for water, waiting for electricity, waiting for clinics that never open. We are alive, but barely.”

In Mqanduli, Asanda echoed similar sentiments:

“We waited for years for a water project that never came. The clinic is empty, and when I ask why, they say, ‘Patience.’ But patience does not feed the children. My heart feels heavy every day. Even my tears are tired.”

Most participants described a cumulative sense of political betrayal, a temporal dimension of slow violence that insidiously shaped mental and physical health. Scarcity, then, is not just poverty; it is a deliberate neglect whose effects are embodied, relational, and gendered.

### 3.7. Gendered Resource Insecurity and Survival Strategies

Women reported diverse strategies to navigate WEF insecurity. In Lorentzville, 15 out of 23 participants described informal work, transactional sex, or occasional sex as survival strategies to secure food, rent, and necessities for children. Ruth, 24, explained:

“Sometimes I skip meals so the children can eat. When there is nothing left, I try to do people’s hair or sell small things. When that fails, I borrow, but borrowing also has its shame. Every day I wake up thinking, How will we survive today?”

In Mqanduli, subsistence farming, food borrowing, and rotating credit groups (stokvels) provided limited buffers. Mam’ Dingiswayo, 55, reflected:

“People used to share maize or help with chickens; now everyone is just trying to survive. There is no abundance left, only what you can get your hands on today.”

Scarcity was relational, shaping mobility, social ties, and household stability. Mental health consequences were widespread. Anxiety, insomnia, headaches, and depressive symptoms were common. Men also reported distress when unable to fulfill provider roles, often expressed through substance use or aggression, compounding IPV risk.

### 3.8. Survival Sex, Transactional Sex, and Reproductive Vulnerability

For many women in Lorentzville and Mqanduli, scarcity pushed them into survival strategies involving sexual exchanges, ranging from occasional sex to transactional arrangements or full-time sex work. These strategies were rarely framed as choices but as necessities driven by hunger, rent, or the basic needs of children. Zelda, 33, a mother of three, described her experience:

“I never planned to sell my body, but my children were crying for food. One night, I sat with my baby on my lap and thought, if I don’t do this, he will sleep hungry. The client offered enough to buy food for a week. I said yes, but inside, I hated myself. The hunger made the plan for me, not me.”

Transactional sex was often coercive, with clients pressuring women to forgo condoms or demanding additional sexual acts in exchange for payment. Malaika, 30, explained:

“If he says no condom and offers more money, I cannot say no. I need the money for rent, for school fees, and for food. I feel trapped; my body is not mine. Sometimes I leave scared, sometimes I cry alone. I hate this life, but I cannot stop.”

Occasional sex, such as short-term exchanges of intimacy for food, electricity, or small sums of money, was similarly fraught. Tebogo, 26, recalled:

“If I refuse, the landlord cuts my electricity. If I accept, I feel disgusted with myself. I try to act normal in front of the children, but at night, I cannot sleep. I feel my body is a weapon against my own dignity.”

Access to reproductive health services was severely constrained. Most participants reported that contraception was inaccessible due to bureaucratic barriers, cost, or moral policing. Grace, a Malawian woman, described a failed attempt to obtain contraceptive pills:

“The clinic said I need papers. I went to buy pills from someone else, but they made me sick. Next month I’m going to get pregnant again. There was no choice. I could not go back to the clinic; they would judge me.”

Abortion, while legal in South Africa, was similarly out of reach for many. Women relied on informal methods, such as herbal teas or unsafe procedures, often experiencing trauma, infection, and anxiety. Nontombi, 22, recounted:

“I tried to get an abortion at the clinic, but they said I was too young. I ended up using an herbal remedy my aunt gave me. I bled for days. I was scared to go to the hospital; I felt they would blame me, call me irresponsible. I felt alone, trapped, and sick.”

Across participants, survival sex and transactional arrangements were entangled with IPV, mental health challenges, and reproductive vulnerability. The coercion, whether from partners, clients, or structural conditions, constrained women’s autonomy and exposed them to sexual, emotional, and health risks. These experiences highlight how the intersections of scarcity, gendered labor, and reproductive governance push women into dangerous survival strategies, reinforcing cycles of structural violence and embodied inequality.

## 4. Discussion

### 4.1. Gendered Survival Strategies and Emotional Burdens Under Scarcity

In Mqanduli, peri-urban women relied on gardening, food borrowing, and collective initiatives such as stokvels and communal gardening. These practices provided limited material and emotional support but were insufficient to fully mitigate the impact of water, energy, and food scarcity. Gendered labor burdens were particularly pronounced, with women performing intensive physical work while remaining largely excluded from decision-making processes regarding land, water, and household resources. Mental health stressors, such as exhaustion, anxiety, and depressive symptoms, were compounded by reproductive challenges, including menstrual infections and miscarriages, highlighting the interconnectedness of scarcity, reproductive health, and psychological well-being.

Across both contexts, the findings highlight that survival strategies were highly gendered, socially mediated, and embedded within fragile networks of solidarity. While informal support networks and collective care practices provided essential relief, these were often temporary and vulnerable to the pressures of scarcity and competition. Importantly, the data indicate that scarcity functions not only as a material condition but as a driver of embodied and emotional stress, shaping daily decision-making, reproductive autonomy, and social relations. This synthesis reinforces the central argument of the paper: WEF insecurity is a structural determinant of both mental and reproductive health, with impacts mediated by gender, social position, and access to resources.

### 4.2. Embodied Scarcity: Reproductive, Sexual, and Mental Health Impacts

Women’s experiences of water, energy, and food scarcity in Lorentzville and Mqanduli reveal the intimate entanglement of material deprivation with reproductive, sexual, and mental health vulnerabilities, highlighting the embodied and emotional dimensions of scarcity that are often overlooked in conventional WEF nexus approaches. Most participants described a daily existence shaped by anticipation, worry, and moral labor: queuing for unreliable water sources, navigating load-shedding and illegal electricity cuts, and rationing food while simultaneously managing household responsibilities. These experiences demonstrate that scarcity is not simply an environmental or logistical problem but a deeply gendered, affective, and bodily phenomenon. In Lorentzville, women described the strain of collecting water before sunrise and facing food insecurity in their households, while in Mqanduli, long distances to boreholes, transport costs, and unpredictable rainfall compounded exhaustion. The majority of participants emphasized that this constant state of negotiation and endurance left them physically and emotionally drained, reflecting what has been theorized as the slow violence of structural neglect [18,44]. These ethnographic insights provide empirical grounding supporting the argument that the WEF nexus literature remains largely technocratic, emphasizing resource efficiency while failing to account for lived experiences, gendered labor, and reproductive health outcomes.

The cumulative effects of scarcity on reproductive and sexual health were profound. Many women described obstructed access to reproductive services, which they experienced as both a bodily and social burden. Limited access to clean water and sanitation further constrained menstrual hygiene management, while chronic stress and material deprivation shaped how women navigated contraceptive use and sexual relationships. Access to contraception and abortion was similarly restricted, especially for undocumented women or those without identity documents, who frequently encountered bureaucratic barriers, stigma, or moral policing in clinics. This institutional exclusion reflects reproductive governance, where administrative and cultural practices regulate women’s bodies and limit their reproductive autonomy [45,46,47,48,49].

Unsafe, self-managed abortion practices were common, often in unsanitary conditions, highlighting the intersection of reproductive risk and structural neglect [50,51]. Globally, similar patterns are evident: in urban informal settlements in India, Brazil, and parts of Southeast Asia, inadequate water, sanitation, and energy access correlate with reproductive health vulnerabilities, unsafe abortions, and psychosocial distress [52,53]. In Sub-Saharan Africa, studies from Kenya, Malawi, and Tanzania similarly demonstrate that women navigating resource-poor environments face constrained reproductive autonomy, linking scarcity, sexual vulnerability, and maternal health risk [54,55,56]. These parallels suggest that reproductive vulnerability under scarcity is not an isolated South African phenomenon but a widespread manifestation of structural inequity and neglect.

### 4.3. Scarcity, Violence, and Reproductive Governance

Scarcity and intimate partner violence (IPV) intersect in a continuum of vulnerability that shapes daily life. Economic deprivation often escalated male frustration into violence, with food and resource shortages directly linked to abuse. Many women reported transactional and occasional sex as survival strategies to secure food, rent, or electricity, navigating coercion from partners or clients while simultaneously lacking access to contraception. In Lorentzville, participants described negotiating survival sex under duress, where refusing to comply with clients’ demands risked cutting off essential resources. These narratives reflect the gendered burden of scarcity: women’s bodies become both sites of labor and arenas of negotiation under coercive pressures. Similar dynamics have been documented in Sub-Saharan Africa, where women in resource-insecure households frequently engage in survival sex or sex work to meet basic needs, experiencing heightened exposure to sexual violence and reproductive risk [55,56,57,58]. Internationally, research in South and Southeast Asia confirms that survival sex and transactional sexual labor are adaptive strategies imposed by structural deprivation rather than moral failings, producing intersecting risks for health and wellbeing [59].

### 4.4. Mental Health as an Expression of Structural Violence

Mental health outcomes are intimately tied to scarcity, reproductive vulnerability, and IPV. Women used culturally specific idioms, “thinking too much,” “a womb that doesn’t rest,” “a heart that no longer feels”, to describe stress, anxiety, and emotional exhaustion, highlighting the embodiment of structural violence. Chronic worry over food, water, electricity, and children’s well-being translated into sleeplessness, headaches, and somatic distress, often compounded by reproductive trauma such as miscarriage, unsafe abortion, or menstrual pain. Men, too, reported psychological strain, particularly when failing to fulfill socially prescribed provider roles, which often manifested in substance abuse and IPV, reinforcing the interconnections of scarcity, gendered expectations, and emotional harm. These findings resonate with global evidence linking resource insecurity and deprivation to mental health burdens in low-income contexts, highlighting that mental distress is not merely an individual pathology but an index of structural inequality [60].

### 4.5. Fragile Solidarities and the Limits of Collective Care

Women’s social networks emerged as essential, though fragile, strategies of resilience. Informal care networks, including rotating cooking duties, shared water collection, and savings clubs (stokvels), provided critical emotional and material support. Such acts of solidarity reflect Tronto’s [60] concept of political care, wherein women sustain life ethically in the absence of state support. However, these networks were precarious, often strained by competition for limited resources or fatigue. This aligns with South African studies indicating that community solidarity mitigates but cannot replace systemic neglect [61,62], and with international research showing that collective survival strategies are both morally sustaining and temporally limited [63,64].

### 4.6. Re-Envisioning the WEF Nexus Through a Gendered and Political Lens

Taken together, these findings demonstrate that the WEF nexus cannot be meaningfully analyzed without attention to gendered labor, reproductive health, mental well-being, and social dynamics. Scarcity in Lorentzville and Mqanduli illustrates that water, energy, and food insecurities are inseparable from embodied politics: waiting for resources, managing household care, navigating reproductive coercion, and enduring emotional strain constitute the lived reality of scarcity. These experiences reinforce critiques that existing WEF frameworks remain technocratic and largely abstract, failing to integrate the lived experiences of the most vulnerable. Women’s narratives highlight that resource insecurity is deeply political, reflecting historical inequalities, infrastructural neglect, and patriarchal governance, while shaping bodily autonomy and mental health.

### 4.7. Policy and Practice Implications

Policy and practice must therefore adopt a holistic understanding of the WEF nexus, integrating gender, reproductive justice, mental health, and IPV into resource management. Access to water, energy, and food is inseparable from reproductive healthcare, safe abortion, contraception, and protection from IPV. Structural interventions must address not only technical delivery but also bureaucratic barriers, moral policing, and social marginalization, ensuring that survival does not require transactional or coerced sexual labor.

Finally, this study emphasizes that women’s endurance should not be romanticized as resilience alone. While informal care, collective resource sharing, and adaptive strategies reflect remarkable agency, they simultaneously underscore systemic failure. Women’s “making a plan” to survive, negotiating scarcity and coercion, and supporting one another in contexts of chronic deprivation illustrate both the ethical and political dimensions of everyday survival. The silent scars left by scarcity, emotional exhaustion, reproductive trauma, coerced sexual labor, and constrained autonomy highlight the urgent need for integrated, justice-oriented approaches to resource management that recognize the entanglement of environmental, social, and bodily wellbeing. In sum, achieving reproductive and resource justice in South Africa, and in comparable global contexts, requires a recognition of scarcity not as a technical challenge but as a deeply embodied, gendered, and political phenomenon, demanding interventions that restore dignity, autonomy, and care.

## 5. Conclusions and Recommendations

This study examined how water, energy, and food scarcity shape women’s reproductive and mental health in Lorentzville and Mqanduli. The findings demonstrate three key points: First, scarcity directly contributes to menstrual challenges, miscarriage, unsafe abortion, and chronic stress, confirming the strong link between resource insecurity and health. Second, women’s survival strategies, including informal work, borrowing networks, and transactional or survival sex, show how scarcity limits autonomy and exposes them to coercion and violence. Third, historical and institutional inequities intensify these vulnerabilities, demonstrating that the water, energy, and food nexus is closely connected to gender, power, and social justice.

Together, these findings show that the water, energy, and food nexus can be strengthened by placing women’s lived experiences at its center and by recognizing resource insecurity as both a structural and health-related issue.

### 5.1. Recommendations

Policy and practice must reflect the interconnectedness of resource insecurity, reproductive health, and mental well-being. This study recommends the following:

- Integrate water, energy, and food policy with reproductive health, mental health, and protection from intimate partner violence.
- Ensure equitable access to contraception, safe abortion, and reproductive healthcare for all women, including undocumented migrants and residents of informal settlements.
- Adopt rights-based approaches to sex work to reduce coercion and improve safety for women who rely on transactional or survival sex to meet basic needs.
- Strengthen social protection measures such as cash transfers, food support, and community-based resource programs to reduce the material pressures that drive high-risk coping strategies.
- Embed mental health support into primary healthcare and community structures to address stress, anxiety, trauma related to intimate partner violence, and reproductive distress.

### 5.2. Limitation

Despite the strength and depth of the qualitative evidence presented, this study focused on women in two South African communities, which may limit the transferability of the findings to settings with different socio-economic or cultural contexts. The study addressed sensitive topics such as transactional sex and intimate partner violence, and these were based on self-reported accounts that may be shaped by stigma, fear, or social desirability. Certain groups, including adolescent girls who were not part of household networks and women with disabilities, were not fully represented in the sample. These limitations highlight the need for future research that includes broader and more diverse participants in order to deepen and extend the insights generated here.

### 5.3. Final Statement

Addressing water, energy, and food scarcity is not only a technical matter but an ethical and political priority. Scarcity, reproductive vulnerability, and violence are interconnected forms of structural inequality. Achieving reproductive and resource justice in South Africa requires coordinated policies that recognize how environmental, social, and bodily realities intersect in women’s lives. True justice requires rebuilding material, social, and institutional infrastructures of care that respect women’s rights to health, autonomy, and dignity.

## Figures and Tables

**Table 1:** Socio-demographic characteristics of women in Lorentzville (*n* = 23).

Participant (Pseudonym)	Age	Marital Status	Migration or Citizenship Status	Education Level	Main Income or Survival Strategies
Nelly	55	Widowed	South African, internal migrant	Incomplete secondary	Care work, borrowing networks
Maria	43	Separated	South African citizen	Completed secondary	Informal work, savings clubs
Josephine	41	Single mother	South African citizen	Some secondary	Informal work, shared cooking
Grace	34	Single	Malawian, undocumented	Some secondary	Piece jobs, occasional sex, informal trade
Phindile	32	Partnered	South African citizen	Incomplete secondary	Dependent on partner, occasional work
Tebogo	26	Single mother	South African citizen	Completed secondary	Occasional sex for electricity access
Zelda	28	Single mother	South African citizen	Incomplete secondary	Transactional sex, informal jobs
Malaika	30	Single	Migrant from Zimbabwe	Incomplete secondary	Occasional sex, informal vending
Amina	36	Married	Migrant from Mozambique	Incomplete secondary	Domestic work, food rationing
Thoko	45	Partnered	South African citizen	Some secondary	Informal selling, borrowing
Thembi	39	Partnered	South African citizen	Lower secondary	Piece jobs
Sandra	27	Single	South African citizen	Some secondary	Informal hair braiding
Ruth	24	Single	South African citizen	Completed secondary	Occasional sex, caregiving work
Nomvula	50	Married	South African citizen	Primary education	Care work
Lindiwe	33	Separated	South African citizen	Some secondary	Renting rooms, stokvel
Patricia	29	Single	Migrant from Lesotho	Incomplete secondary	Piece jobs
Busi	38	Partnered	South African citizen	Primary education	Domestic work
Chipo	35	Migrant	Zimbabwean	Some secondary	Street vending
Angeline	31	Single	South African citizen	Completed secondary	Informal trading
Zodwa	48	Married	South African citizen	Primary education	Care work
Refiloe	22	Single	South African citizen	Matric	Piece jobs
Amanda	30	Partnered	South African citizen	Incomplete secondary	Domestic work
Farai	40	Migrant	Zimbabwean	Some secondary	Informal trade

**Table 2:** Socio-demographic characteristics of women in Mqanduli (*n* = 13).

Participant (Pseudonym)	Age	Marital Status	Citizenship	Education	Main Income or Survival Strategies
Asanda	33	Married	South African	Some secondary	Casual labor, food borrowing
Zanele	36	Married	South African	Primary	Communal food sharing, gardening
Nozipho	29	Married	South African	Primary	Water collection, household labor
Thandi	25	Married	South African	Completed secondary	Casual labor, borrowing
Nontombi	22	Single	South African	Secondary	Seeking contraception, informal work
Mam’ Dingiswayo	55	Widowed	South African	Primary	Small-scale maize cultivation
Vuyiswa	40	Married	South African	Primary	Borrowing, rotating credit groups
Sindiswa	30	Partnered	South African	Some secondary	Household care work
Ayanda	19	Single	South African	Secondary	Occasional farm labor
Nobuhle	27	Partnered	South African	Secondary	Gardening, borrowing
Zikhona	31	Married	South African	Primary	Informal domestic tasks
Lungile	45	Married	South African	Primary	Small livestock care
Phelokazi	50	Married	South African	Primary	Collecting water, small crop planting

**Table 3:** Overview of themes identified through reflexive thematic analysis.

Theme	Description	Illustrative Subthemes	Example Quotations
Living within scarcity	Emotional and bodily strain of navigating daily shortages	Cognitive fatigue, somatic distress, worry, exhaustion	“My mind is burning…”, “Even my tears are tired.”
Fragile solidarities and collective care	Collective strategies that offer relief but remain unstable	Mutual aid, food sharing, emotional support, conflict under scarcity	“We laugh in line so we do not cry…”
Gendered labor and emotional exhaustion	Invisible emotional and material labor carried by women	Household planning, care burdens, anxiety, exclusion from decisions	“If I stop planning, the children will go hungry…”
Reproductive pain and constrained autonomy	How scarcity limits reproductive health and safety	Menstrual challenges, miscarriage, unsafe abortion, clinic barriers	“I bleed and it burns…”, “They said I need papers…”
Scarcity, violence, and despair	Intersections of deprivation and interpersonal violence	IPV, coercion, male frustration, emotional collapse	“I stay because if I leave, the children will starve…”
Survival sex and reproductive vulnerability	Sexual exchanges as survival strategies	Occasional sex, transactional sex, coercion, lack of contraception	“Hunger made the plan for me, not me…”

## Data Availability

Due to the sensitive and confidential nature of the qualitative data, the minimal dataset supporting the conclusions of this article is available from the corresponding author on reasonable request.
